# Smart thin porous calcium phosphate coatings for local antibiotic delivery

**DOI:** 10.1186/s13104-025-07453-3

**Published:** 2025-09-01

**Authors:** Long-Quan R. V. Le, Maria Carolina Lanzino, Anika Höppel, Mirjam Rech, Sofia Dembski, Andreas Killinger, Bianca Riedel, Michael Seidenstuecker

**Affiliations:** 1 G.E.R.N. Center of Tissue Replacement, Regeneration & Neogenesis, Department of Orthopedics and Trauma Surgery, Faculty of Medicine, Albert-Ludwigs-University of Freiburg Medical Center, Hugstetter Straße 55, 79106 Freiburg, Germany; 2 Institute for Ceramic Materials and Technologies (IKMT), University of Stuttgart, 70569 Stuttgart, Germany; 3 Department for Functional Materials in Medicine and Dentistry, University of Würzburg, 97070 Würzburg, Germany; 4 Fraunhofer Institute for Silicate Research ISC, 97082 Würzburg, Germany

**Keywords:** Coatings, HVSFS, β-TCP, Vancomycin, Supraparticles

## Abstract

**Objective:**

Implant failure after arthroplasty, primarily due to aseptic loosening or periprosthetic joint infection, remains a significant clinical problem. Bioactive ceramic coatings, such as β-tricalcium phosphate (β-TCP), enhance osseointegration and may reduce the risk of aseptic loosening. At the same time, localized antibiotic release from the implant surface represents a promising strategy to prevent early bacterial colonization. The aim of this study was to evaluate the feasibility of incorporating the heat-sensitive antibiotic vancomycin (VAN) into β-TCP coatings using high-velocity suspension flame spraying (HVSFS).

**Results:**

We successfully embedded VAN into β-TCP coatings by preparing suspensions containing VAN-loaded supraparticles as feedstock for the HVSFS process. High-performance liquid chromatography analysis confirmed that VAN maintained its chemical integrity during spraying, with spectra comparable to untreated controls, indicating no thermal degradation. The resulting multifunctional coatings therefore combined the osteoconductive potential of β-TCP with the antibacterial activity of VAN. These findings demonstrate that HVSFS is a viable technique for producing bioactive coatings that simultaneously promote bone integration and enable local antibiotic delivery, offering a potential strategy to mitigate both aseptic loosening and infection risks in arthroplasty.

## Introduction

Total joint arthroplasty (most commonly hip and knee replacement) is a common surgical procedure in which damaged joint surfaces are removed and replaced with prosthetic components (metal, ceramic, and/or polyethylene) to relieve pain, restore function, and restore the biomechanics of the joint. The surgery usually involves exposing the joint, removing diseased bone and cartilage, preparing the bone surfaces, and inserting and fixing modular components designed for long-term load transfer and biological integration (osseointegration) with the host bone. Successful arthroplasty depends on the precise positioning of the implant, stable initial fixation, and a favorable biological interface that allows bone ingrowth or permanent cement fixation [1]. Despite generally excellent results, implant failure after arthroplasty remains a major clinical challenge. There are two main types of failure: septic, which is periprosthetic joint infection (PJI), and aseptic, which is mechanical or biological. Aseptic failure can include aseptic loosening, particle-induced periprosthetic osteolysis, instability/dislocation, implant fracture, malalignment, and pain of unknown cause. The importance of these mechanisms varies depending on the joint type, time since implantation, and implant design. Infections and instability commonly cause early failure, while aseptic loosening and osteolysis commonly cause late failure. Large series and registry analyses continue to identify aseptic loosening and instability as the main causes of revisions after total hip arthroplasty. However, infections have become an increasingly important cause of knee joint revisions in some newer cohorts [2–6]. Overcoming these problems requires a combined approach: improving osseointegration while preventing bacterial infections. Recent research has demonstrated the potential of biocompatible materials to improve implant-bone integration [7, 8]. In thin calcium phosphate (CaP) coatings, the balance between the degradation rate of the coating and the kinetics of bone regeneration is crucial. In resorbable ceramics, this process should be synchronized - ideally, the coating is gradually resorbed by the body and replaced by newly formed bone [9, 10]. The study is part of the project “Thin resorbable coatings for optimizing osteointegration while preventing infection”. The focus is on the development of thin, porous, and bioresorbable CaP coatings with antibacterial properties, which are applied using high-velocity suspension flame spraying (HVSFS) [11, 12]. An important strategy for increasing porosity and functionality is the incorporation of supraparticles into the layer matrix [13]. These supraparticles, made of nanoparticle (NP) building blocks and loaded with Copper (Cu) as in our previous works [11, 14, 15] as in the present case, with antibiotics, serve as both structural and antibacterial components. To incorporate supraparticles into the matrix, the radial injection of the suspension right after the nozzle can be used, as described in [14]. This allows thermally sensitive materials to be sprayed because their dwell time in the flame is reduced, meaning the material particles are exposed to less thermal stress. After implantation, the embedded supraparticles gradually release Vancomycin (VAN) as the coating degrades. This design aims to achieve high initial VAN release to prevent biofilm formation at an early stage, followed by sustained antibacterial activity throughout the healing phase - until the coating is completely replaced by new bone tissue. Stigter et al. [16] have also produced CaP layers with antibiotics on titanium plates, but using precipitation. Schmidmaier et al. [17] coated K-wires made of titanium using dip coating with poly(D, L-lactide) and growth factors or antibiotics [18].

The study’s objective is to determine if vancomycin (VAN) is susceptible to degradation during the HVSFS of β-TCP coatings. VAN is embedded in β-TCP supraparticles and deposited radially with the axially injected β-TCP starting material. This protects the VAN from high temperatures and enables sustained release. These results lay the groundwork for developing HVSFS-based β-TCP coatings that combine rapid osseointegration with long-lasting antibacterial protection, thus combating the primary causes of implant failure.

## Materials and methods

The supraparticles were produced and the titanium plates coated in the same way as described in our previous work [11, 13, 14, 19]. The only difference is the use of Vancomycin instead of Cu ions.

### VAN-doped supraparticles

#### Materials for supraparticle synthesis and coating process

β-TCP, 97.2%, Chemical Specialist Budenheim, Budenheim, Germany), solution styrene-butadiene rubber (SSBR, 15 wt% in H_2_O, Targray, Kirkland, QC, Canada), phosphonate based dispersion agent (Zschimmer and Schwarz, Lahnstein, Germany) and hydrocolloid (Zschimmer and Schwarz, Lahnstein, Germany). Vancomycin hydrochloride was purchased from Hikma Pharma GmbH (Martinsried, Germany).

#### Preparation of VAN-doped β-TCP supraparticles (TCPVAN particles)

For spray-drying preparation, commercial β-tricalciumphosphate microparticles (TCP) were initially milled in deionized (DI) water at a 1:1 ratio for 1 h at 700 rpm using a planetary micro mill (PULVERISETTE 7 premium line, FRITSCH, Idar-Oberstein, Germany). Subsequently, an aqueous suspension was prepared containing 30 wt% of the milled TCP and 0.25 wt% vancomycin (VAN), relative to the β-TCP content. To this suspension, solution-styrene butadiene rubber (SSBR) was added as a binder at 10 wt% relative to the total TCP + VAN mass. The resulting mixture was stirred for 1 h and then spray-dried under the following conditions: inlet temperature (T_inlet_) of 130 °C, aspirator setting at 100%, and pump rate at 15%.

### Suspensions and coating deposition

Subsequently, upon the identification of optimal parameters, as shown in previous works [11, 14], VAN-doped β-TCP coatings were applied to titanium (Ti) grade 2 substrates (10 × 10 × 3 mm, ARA-T Advance GmbH, Dinslaken, Germany). While the β-TCP serves as the primary constituent for forming the matrix of the resulting coating, the VAN-doped supraparticles, acting as secondary phase, are meant to confer antibacterial properties to the coatings. Additionally, they are expected to increase the microporosity of the coatings. In fact, the supraparticles are not expected to be completely molten during the HVSFS process. As a result, the space between the nanoparticles (NPs) will be retained, creating micro- or nanoporosity [11, 14].

#### Suspensions

Water-based suspensions were employed for the deposition of bioconductive coatings via spraying. β-tricalcium phosphate (β-TCP) raw powder was gradually introduced into a mixture of deionized (DI) water containing two stabilizing agents: 2 wt% of a hydrocolloid and 3 wt% (solid content) of a phosphonate-based dispersing agent, under continuous stirring. For initial optimization trials, β-TCP concentrations of 5 wt% and 10 wt% were tested to modulate the coating porosity. The β-TCP powder used had a particle size distribution with d50 ≤ 10 μm. For the final vancomycin (VAN)-doped coatings, two separate suspensions were prepared: one for the matrix layer, containing only β-TCP, and another for the secondary phase. The matrix suspension was composed of 5 wt% β-TCP prepared as described above. For the secondary phase, 3 wt% VAN-loaded supraparticles were dispersed in DI water with the same stabilizing agents and concentrations as used in the β-TCP suspension.

#### Coating deposition

The coating deposition was carried out as described elsewhere with a radial injection [11, 14, 15]. A modified Top Gun G system (GTV wear protection, Luckenbach, Germany) was used for this purpose. The spray gun was mounted on a six-axis robot to perform a controlled meandering motion with an offset of 3 mm and a spray distance of 120 mm. Ethylene (C_2_H_4_) and oxygen (O_2_) were used for combustion. The coatings were applied to Ti grade 2 substrates. All substrates were sandblasted with F60 corundum at a pressure of 4 bar prior to coating. For the coating process, the suspensions previously described were used as feedstock material. The matrix material was β-TCP, which was injected axially into the combustion chamber. The VAN-doped supraparticles suspension was instead injected radially into the flame right after the nozzle of the spray gun, as described in previous works [14, 20].

### VAN release

The VAN-coated titanium samples were incubated in a release test with 5 ml of bidestilled water at 37 °C in an incubator. After 2 weeks, the solutions were removed and analyzed using HPLC (Shimadzu CBM-20 A, CTO-20AC, DGU-20A5R, LC-20ADXR, Reservoir Tray, RF-20 A, SIL-30AC, SPD-M20A IVDD, Kyoto, Japan; Macherey-Nagel precolumn EC 4/3 Nucleodur 300-5 C4ec, column EC 250/3 Nucleodur 300-5 C4ec, Duren, Germany). A calibration curve of 1–50 µg/ml was prepared and examined together with the released vancomycin. Before measurement, all samples underwent sterile filtration with a 0.2 μm pore size. For VAN analysis, HPLC was conducted at a temperature of 25 °C, with a running time of 10 min and a flow rate of 0.66 mL/min. The mobile phase consisted of ACN and 10 mM NH_4_H_2_PO_4_ (Sigma-Aldrich, now Merck, Darmstadt, Germany), pH 2.2, adjusted with phosphoric acid, in a ratio of 12:88. The area under the curve was measured at a wavelength of 198 nm. The aim was to clarify if the vancomycin is damaged by the coating process.

## Results

**Fig. 1:**
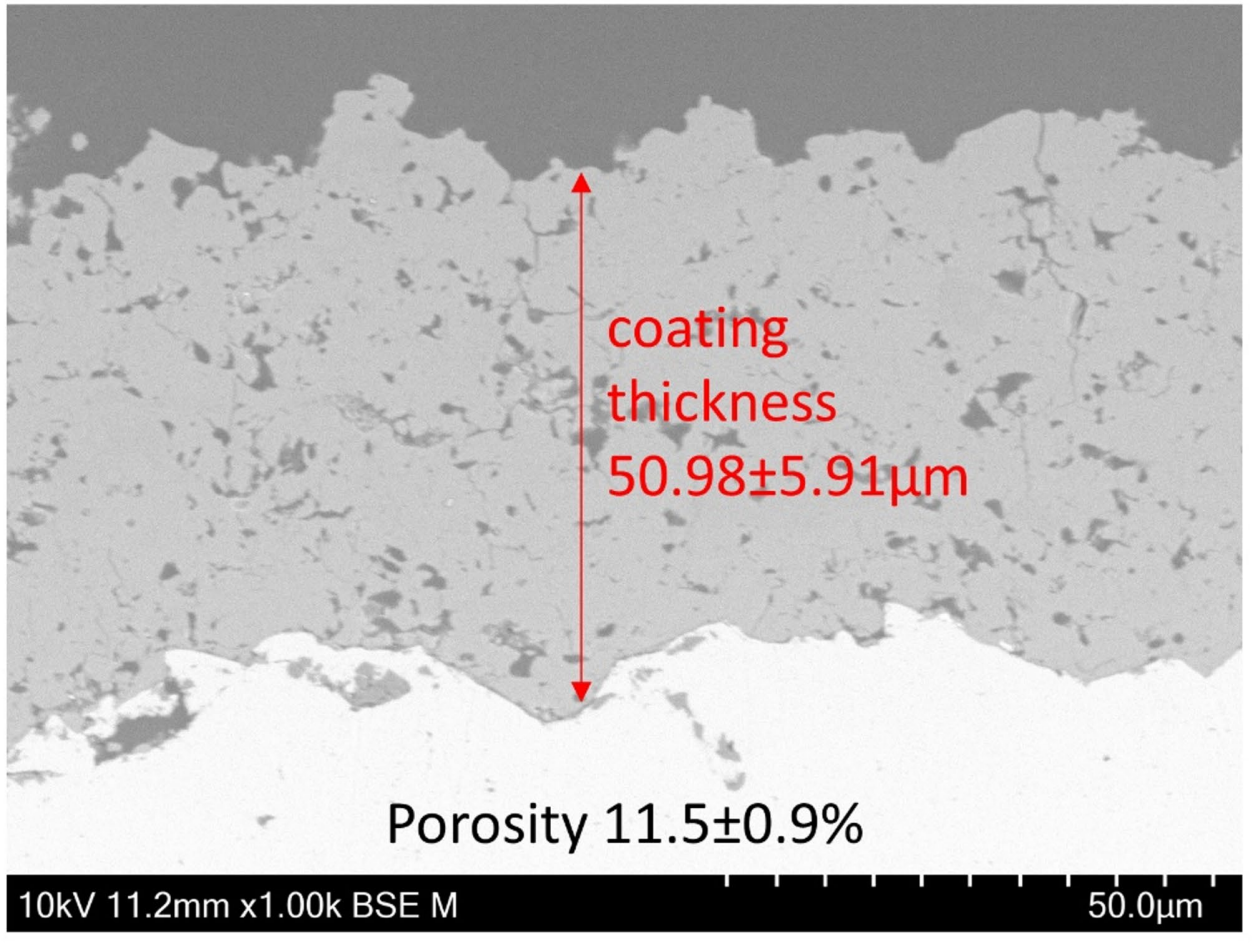
Scanninc electron microscopy (SEM) image showing the VAN-doped β-TCP coating; including information on thickness and porosity of the coating, magnification 1000x, scalebar 50 μm

VAN-doped β-TCP coatings with a thickness of 50.98 ± 5.91 μm and a porosity of 11.5 ± 0.9% were fabricated by HCSFS process (see Fig. 1).

**Fig. 2:**
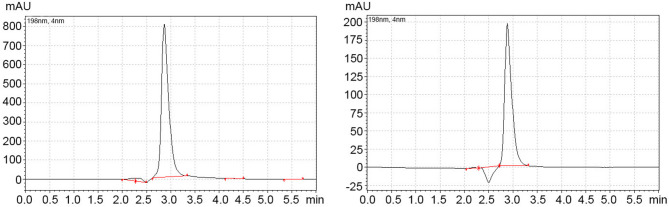
Comparison of VAN peaks by HPLC: left: freshly applied calibration curve; right: release test after HVSFS and 2 weeks incubation

HPLC analysis revealed no thermal decomposition of VAN attributable to the HVSFS process. The VAN protected by the supraparticles showed no signs of decomposition. The peaks were identical to those of the calibration curve. The only difference is due to the different VAN concentrations, which are reflected in different peak heights (see Fig. 2).

**Fig. 3:**
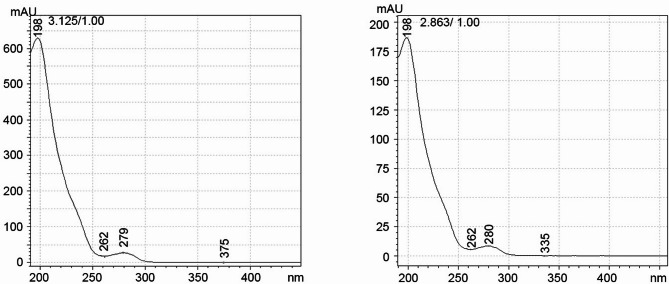
Comparison of the VAN spectra from HPLC: fresh (left) versus originating from HVSFS (right)

No additional peaks were detected in the spectra either. Figure 3 shows a comparison of the VAN spectra: fresh versus released from coating after 2 weeks incubating in double dest. water. Again, the only difference is the peak height/area due to the different concentrations.

**Fig. 4:**
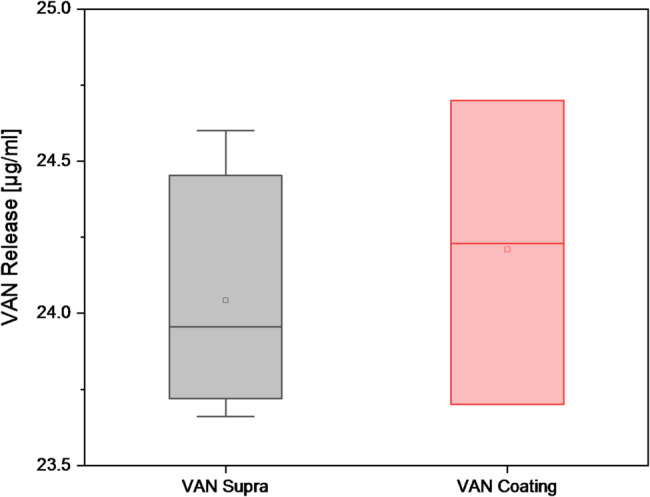
Box plot of VAN releases for different types of coating after 2 weeks by HPLC

After incubation for 2 weeks at 37 °C, the release of VAN from the supraparticles within the β-TCP coating was 24.04 ± 0.40 µg/mL. The release of VAN from the coating itself was 24.21 ± 0.5 µg/mL. No significant difference was found between the two different releases. The p-value determined by the Tukey test was 0.642. Figure 4 shows the box plots for both VAN releases after 14 days.

## Discussion

The thermal stability of vancomycin has been investigated using various analytical methods. Thermogravimetric analysis shows that crystalline vancomycin undergoes an initial endothermic event at around 40 °C due to dehydration and oxidative demethylation. A second transition occurs at approximately 169 °C, which corresponds to the melting of its amorphous phase [21]. A stability study evaluating concentrated vancomycin solutions exposed to 100 °C for up to 60 min showed no significant loss of concentration in 5% glucose or 0.9% NaCl solutions, indicating robust short-term thermal resilience [22]. Conversely, studies stressing vancomycin solutions under heat in combination with acidic or alkaline media - such as exposure at 80 °C for 300 min - resulted in approximately 20% degradation. This demonstrates that enhanced thermal degradation occurs under extreme pH and prolonged heating conditions [23]. Furthermore, rapid degradation was observed when vancomycin solutions were exposed to strong acidic or basic environments (0.5 M HCl or NaOH) at elevated temperatures (60–90 °C). At 80 °C, the half-life decreased significantly, and calculated rate constants confirmed accelerated breakdown via pseudo-first-order kinetics [24]. The spectra from the HPLC measurements clearly show that VAN can withstand the temperatures in the flame during the HVSFS process. Ensom et al. [25] described in their work, that after 18 h at 100 °C, only 58% of the VAN was detectable by HPLC. They also impressively demonstrated degradation peaks of VAN that occurred before and after the main peak. Dolete et al. [26] refer to oxidative decomposition of VAN after reaching a temperature of 150 °C. No degradation peaks were observed in our experiments. However, this is probably due to the very short residence time in the flame. Buss et al. [27] support this theory by demonstrating that the particle residence time (PRT) can be influenced by the geometry of the reaction chamber and the rate of coaxial gas flow. At a temperature of 1000 K and a flow rate of > 400 L/min, the PRT was 0.03 s, and only by increasing the temperature to 1500 K was the PRT reduced to 0.001 s. The advantage of our process, which incorporates VAN into the TCP supraparticles, is thermal shielding. Together with the short residence time in the flame, this results in very good protection against thermal decomposition. We assume that the VAN in the supraparticles did not reach these temperatures at all in the HVSFS and 100% of VAN survived the processing. This is also shown by the unchanged peaks and spectra.

### Limitations and future perspectives

A notable restriction of this study is the utilization of vancomycin (VAN) as the model antibiotic for all coatings. The stability of VAN under short thermal exposure during the HVSFS process is notable, yet other antibiotics with distinct physicochemical properties, such as clindamycin, may manifest varied thermal degradation profiles and drug release behaviors, particularly in the context of incorporation into supraparticles. Furthermore, this investigation was conducted at the culmination of an ongoing project as a proof-of-concept, and the antimicrobial efficacy was not systematically assessed. Consequently, subsequent studies should broaden the array of antibiotics evaluated, assess the impact of supraparticle incorporation on their stability, and incorporate exhaustive antimicrobial performance assays to thoroughly substantiate the clinical viability of HVSFS-derived β-TCP coatings.

## Conclusion

We were able to confirm that the supraparticles are suitable as carriers for antibiotics and can therefore be used to incorporate temperature-protected drugs into implant coatings. We were also able to show that VAN survives the HVSFS process unscathed, and that there is some protection against the high temperatures of the flame.

## Data Availability

The datasets used and/or analyzed during the current study are available from the corresponding author on reasonable request.
